# Extract from endophytic *Fusarium* isolates stimulates seed germination of the host and protocorm development of non-host orchids

**DOI:** 10.1080/19420889.2024.2439798

**Published:** 2024-12-17

**Authors:** Sujit Shah, Mukti Ram Paudel, Bir Bahadur Thapa, Harshita Sharma, Arun Kumar Kashyap, Bhagwan Narayan Rekadwad, Rohit Sharma, Jyotsna Sharma, Bijaya Pant

**Affiliations:** a Central Department of Botany, Tribhuvan University, Kirtipur, Kathmandu, Nepal; b Department of Plant Science and Soil Science, Texas Tech University, Lubbock, TX, USA; c Molecular Biotechnology Laboratory, School of Biotechnology, Institute of Agricultural Technology, Suranaree University of Technology, Nakhon Ratchasima, Thailand; d Department of Biotechnology, Govt. E Raghavendra Rao PG Science College, Bilaspur, India; e Yenepoya Research Centre, Yenepoya (Deemed to be University), Mangalore, India; f National Centre for Microbial Resource (NCMR), DBT-National Centre for Cell Science (DBT-NCCS), Savitribai Phule Pune University Campus, Pune, India; g School of Sciences, SAM Global University, Raisen, India

**Keywords:** *Dendrobium*, fungal extract, *Fusarium*, Indole acetic acid, orchid

## Abstract

We isolated endophytic *Fusarium* strains from the healthy roots, stems, and leaves of *Dendrobium moschatum* to investigate their plant growth-promoting activities in vitro. Subsequently, Indole acetic acid (IAA) was quantified and the *IaaM* gene (responsible for IAA synthesis in fungi) was amplified and sequenced. Finally, a germination assay was performed with seeds of *D. moschatum* and a plant growth assay with protocorms of *Dendrobium longicornu* to test their plant growth-promoting activities. Five *Fusarium* isolates (CDS11, PDL1, PDL3, PDR6, PDR7) were identified in this study. The highest amount (60µgml^−1^) of indole acetic acid was recorded in the PDR7 extract, whereas it was not detected in PDR6 and CDS11. The fungal extracts of isolates PDR6 and PDR7 were highly effective for seed germination by approximately 80% and 90% (respectively) of the host plant. The fungal extract of PDR7 showed a high IAA content and promoted in vitro seed germination of the host (*D. moschatum*) and protocorm development of the non-host (*D. longicornu*). In contrast, IAA content in the fungal extract of PDR6 remained undetected but was effective in both seed germination and protocorm development. Our results demonstrated the potential beneficial application of endophytic *Fusarium* in orchid mass propagation.

## Introduction

Orchids are the most diverse group of plants occurring in all continents, except Antarctica. According to a recent study, Nepal’s diverse ecosystems are home to approximately 501 species of orchids [1]. Due to overexploitation and forest loss, several species are in danger of extinction [1,2]. Orchidaceae has been reported as the second-largest plant family in Nepal [3,4]. *Dendrobium* is the largest genus in the orchid family in Nepal. Twenty-nine *Dendrobium* species have been reported in Nepal [5]. *Dendrobium* species have been extensively studied for their phytochemical, antioxidant, and free radical scavenging properties [6–9]. *Dendrobium moschatum* considered in this study is well-known for its musk-smelling fragrance and its ornamental and therapeutic qualities [10]. It is a common epiphytic orchid with an upright cylindrical stem, uneven bilobed leathery leaves and deep yellow colored flower that grows in the temperate regions of northeastern India, Nepal, and Bhutan [11].

Plant growth-promoting effects of endophytic microbes have been reported in various orchids [12–21]. Indole-3-acetic acid (IAA) is a naturally occurring plant hormone that is generally referred to as auxin. They play a crucial role in plant growth and development. It participates in cell signaling and coordination, cell elongation, cell division, and root initiation and elongation [22]. IAA is produced by two pathways: tryptophan-dependent and tryptophan-independent. The try-dependent pathway plays a critical role in embryonic development, seedling growth, flower and vascular tissue development, and other physiological processes. Several reports have demonstrated the ability of endophytic fungi to use the tryptophan dependent indole-3-acetamide (IAM pathway) for IAA production [23–25]. The first described orchid endophytes were *Fusarium* spp., which used the IAM pathway for IAA synthesis [26]. Non-pathogenic, endophytic *Fusarium* spp. have been consistently reported in orchid species as plant growth promoters, stimulators for seed germination, and immunomodulators for abiotic and biotic stress [4,27]. In this study, we aimed to 1) isolate and identify endophytic fungi from native *Dendrobium moschatum*, 2) quantify the IAA concentrations in fungal extracts, and 3) experimentally test the ability of the extracts to improve germination and plantlet development in the host (*Dendrobium moschatum*) and a non-host orchid (*Dendrobium longicornu*) in vitro.

## Material and methods

### Sample collection and isolation and identification of fungi

In the present study, we selected *Dendrobium moschatum* growing in a natural temperate habitat, Makwanpur District, central hills (Hetauda) of Nepal. Roots were excised from the wild species, wrapped with tissue paper, placed in zipper bags, and transferred to the laboratory. Healthy *D. moschatum* roots, stems, and leaves of *Dendrobium moschatum* were processed for surface sterilization [28]. Roots were gently washed in running water to remove dust particles and then treated with 3% sodium hypochlorite (NaOCl) for 30 s, followed by treatment with 75% ethanol for 1 min. After this step, it was rinsed with sterile distilled water and dried with a sterile paper towel in laminar wood under aseptic conditions. The outer epidermal layer was removed to prevent contamination. The fungi were isolated from sterile plant tissues. The Plant tissues (root, stem, and leaves) were placed in potato dextrose agar (PDA) or Czapek Dox Agar (CDA) medium and incubated for 7 days at 28°C. Fungi grown from plant tissues were isolated and cultured based on their growth patterns and morphotypes.

DNA isolation of DNA the CTAB method. In this regard, 5 g of the plant part was harvested and ground in liquid nitrogen with a pre-chilled mortar and pestle. The entire content was then transferred to an oak ridge tube. CTAB extraction buffer (10 ml of CTAB extraction buffer was added to the powder and the tube was incubated for 1 h with occasional mixing by gently inverting the tubes. After 1 h, 10 ml of chloroform: isoamyl alcohol was added to the tube to separate DNA from other impurities. The upper layer was transferred to a new tube with the help of cut tips to this 0.1 ml of 3 M sodium acetate buffer (pH 5.2). Thereafter, 2 ml of ice-cold ethanol was added. The tube was spun at 12,000 rpm and the supernatant was removed, and the pellet was washed with 70% ethanol and again spun for 10 min at 10,000 rpm. The pellet was dried at room temperature, and 200 μL of TE buffer was added to it along with 10 μL of RNase -A and incubated for 30 min at 37°C. DNA aliquots were stored at 4°C.Polymerase chain reactions (PCR) were performed on a Mini-cycler TM (MJ Research, Reno, NV, USA). PCR Products were analyzed by gel electrophoresis on 1% agarose gels, stained with ethidium bromide, and visualized under UV light. After purification with mini-columns, the purified DNA was directly sequenced. The primers used were ITS1 and ITS4. The PCR was programmed as follows: initial denaturation at 94°C for 5 min. followed by 35 cycles at 94°C for 5 min and 57°C for 1 min and at 72°C for 1 min with a final extension step at 72°C for 10 min. The amplified fragments were separated on 1% agarose gel using 1 × TAE buffer at 80 V for 20 min. and was examined using a gel documentation system.

### Phylogenetic analyses

The generated consensus sequences of ITS and LSU were subjected to BLASTn search against the GenBank database for identifying closely related taxa. Reference sequences of closely related taxa were selected based on NCBI nBLAST. The sequences were automatically aligned in the MAFFT version 7 (http://mafft.cbrc.jp/alignment/server/index.html) using default settings [29] and trimmed using trimAl (https://ngphylogeny.fr/tools/) [30]. Maximum likelihood (ML) and phylogenetic analyses were performed for combined gene datasets ITS was done on MEGA 11 tool [31] under the GTR+GAMMA substitution model and 1,000 bootstrap iterations.

### IAA quantification and gene amplification

Czapek dox agar (CDA) medium (20 mL, pH 6.5) was used to cultivate the fungal isolates, either with or without 1 mg L-tryptophan supplement [32,33]. The inoculated broths were incubated in a shaker incubator set to 25°C and 120 rpm for ten days. Following incubation, they were centrifuged for 10 minutes at 4°C at 12,000 rpm. One milliliter of the supernatant and two milliliters of Salkowski reagent were combined, and the mixture was incubated in the dark for 30 min to measure the amount of IAA produced. The optical density was measured at 530 nm once a pink hue appeared using a UV-VIS spectrophotometer (ChromTech-CT 8200). The IAA concentration in the extract was measured using independently created standard IAA curves (10–100 µg ml^−1^). Three biological replicates were used for each experiment. Amplification of *IaaM* gene was performed as described with primers F’- AGT GAC CAG CCT GCT GAT TTC CCT CG and R’- AAG ATC GCA GCC ATT GAG TTG TGC [26]. The PCR amplifications were performed in Veriti thermal cycler (Applied Biosystems, Singapore), following conditions: initial denaturation at 94°C for 5 min. followed by 35 cycles at 94°C for 5 min and 57°C for 1 min and at 72°C for 1 min with a final extension step at 72°C for 10 min. The amplified fragments were separated on 1% agarose gel using 1 × TAE buffer at 80 V for 20 min. and examined using a gel documentation system [26].

### Germination and protocorm development experiment

The seed germination assay was performed with the seeds of *D. moschatum* whereas protocorm development experiment was done for *D. longicornu*. The fungal extracts were prepared from CDS11, PDL1, PDL3, PDR6 and PDR7. For each of the five isolates, fungal extract solution was prepared from the supernatant of 10-day old isolates inoculated in Czapek broth medium supplemented with 1 mg tryptophan. The broth was centrifuged at 5000 rpm, and the supernatant was filtered through Whatman filter paper grade 1:11 μm (medium flow filter paper). MS medium supplemented with fungal extracts (250 µl per liter) was prepared. The medium was then autoclaved 121°C for 20 min. The surface-sterilized seed pod of *Dendrobium moschatum* was placed on a sterile Petri dish containing sterile filter paper to soak the surface moisture from its surface. The seed pod was then cut longitudinally into two halves using a sterile surgical blade. Microscopic seeds of orchids were scooped out with the help of a sterile spatula and inoculated on full-strength MS medium as a control and medium supplemented with extracts from the five *Fusarium* isolates. Approximately 15–20 seeds were plated in each culture tube, and 15 replicates were established to represent each of the six experimental treatments. Seeds were incubated at 25 ± 2°C under a 16/8 hrs photoperiod for ten weeks. Another experiment was performed using previously grown protocorms of the congeneric orchid *D. longicornu*. Aseptically grown initial-stage protocorms of *D. longicornu* were inoculated on full-strength MS medium as a control and medium supplemented with 2% fungal extract. We prepared six replicates for each of the six treatments. Each replicate vessel contained 15 protocorms, which were maintained at 25 ± 2°C under the range of 500–1000 lux illuminance for 16/8 h (light/dark) photoperiod using white fluorescent tubes (Philips, India) [34]. Germination was recorded at an interval of three weeks for ten weeks. Protocorm development was estimated by recording the number of roots and shoots at an interval of two weeks for seven weeks.

### Data analysis

IBM SPSS 20 was used to analyze the data generated from the IAA quantification experiment and the germination and protocorm growth experiments. IAA concentrations (with or without tryptophan) in the five isolates were compared using one-way Analysis of Variance (ANOVA) with the alpha error level set at *p* ≤ .01 (Tukey’s HSD test). Next, Analysis of Variance (ANOVA) was used to compare the germination percentages in *D. moschatum* and the root and shoot numbers in *D. longicornu* in response to the six experimental treatments. The responses were considered statistically different at *p* ≤ .05. (Tukey HSD test).

## Results

### Isolation and identification of plant growth-promoting fungi from *Dendrobium moschatum*

**Figure 1.**
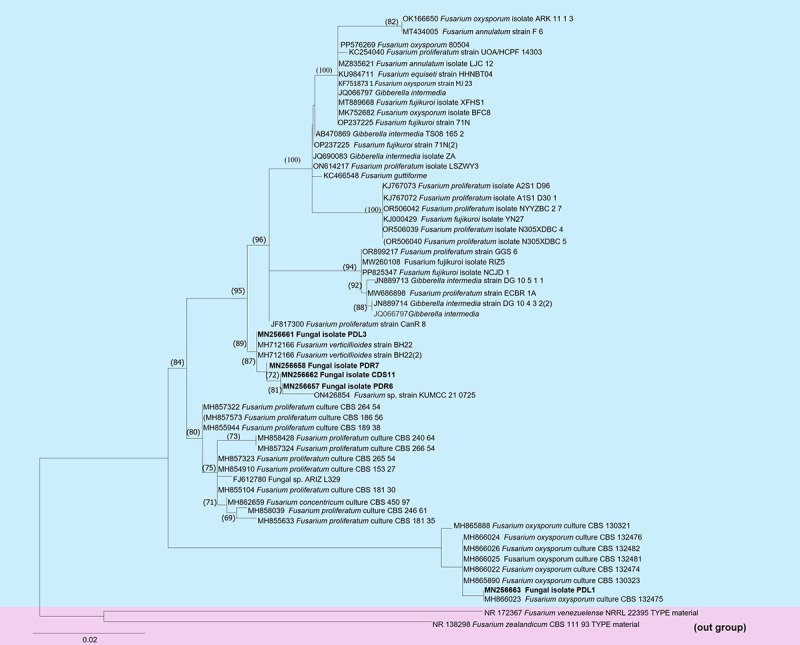
Phylogram generated from maximum likelihood analysis based ITS sequences of the *Fusarium* species. Maximum likelihood bootstrap support values ≥ 69% are shown in the nodes. Newly generated sequences of the isolates CD11, PDL1, PDL3, PDR6 and PDR7 are indicated in bold. The tree was rooted with *Fusarium zealandicum* CBS 11.93 and *Fusarium venezuelense* NRRL 2239.

The fungal isolates PDR6 and PDR7 were obtained from the roots, PDL1 and PDL3 from the leaves, and CDS11 from the stems. All five isolates belonged to the *Fusarium* genus and were identified using internal transcribed spacer sequence (ITS) analysis. The phylogenetic analysis of ITS sequences data of 61 strains of *Fusarium* species of which *Fusarium zealandicum* CBS 11.93 and *Fusarium venezuelense* NRRL 2239 were outgroup as displayed in Figure 1. Our isolates CDS11, PDR6 and PDL3 showed clustered with *Fusarium verticillioides*, whereas PDL1 clustered with *Fusarium oxysporum*. The number of fungal isolates, their identities, and their ITS GenBank accession numbers are listed in Table 1.

**Table 1.** Molecular identification of endophytic fungi isolated from healthy tissues of *Dendrobium moschatum.*

Isolation medium and source tissue	Fungal taxonomy	Isolates	Query Coverage	Identity match (%)	GenBank Accession no. (ITS)
CDS11*	*Fusarium s*p.	2	100%	100%	MN256662
PDL1**	*Fusarium s*p.	2	100%	100%	MN256663
PDL3**	*Fusarium s*p.	3	99%	99.82%	MN256661
PDR6***	*Fusarium s*p.	2	99%	99.64%	MN256657
PDR7***	*Fusarium s*p.	1	99%	99.64%	MN256658

*CDS ‘CD’ represents CDA medium, ‘S’ Stem.

**PDL represents the PDA medium, ‘L’leaf section.

***PDR represents ‘PD’ PDA medium, ‘R’ root.

### Indole acetic acid quantification and gene amplificaiton

**Figure 2.**
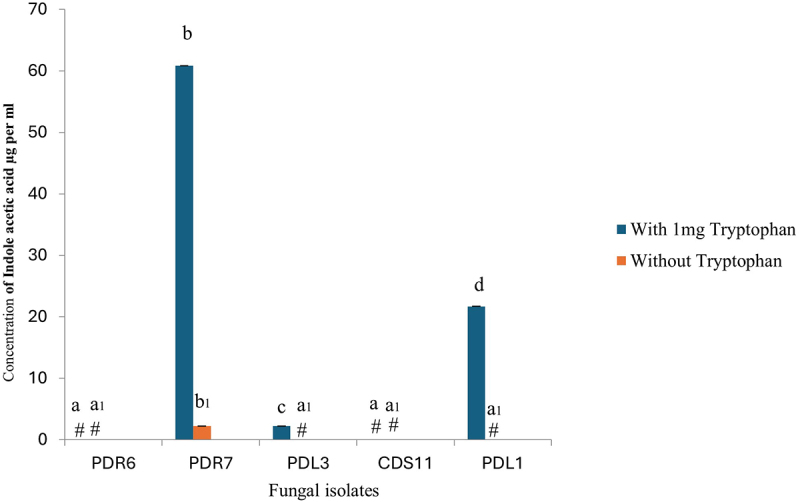
Auxin concentration in fungal extracts with and without added tryptophan. The experiment was repeated three times. Extract from PDR7 had the highest concentration of indole acetic acid (IAA). The bar represents mean ± SE (*n* = 3). Values with different letters are significantly different at *p* ≤ .05 (Tukey test). ‘#’ indicates the absence of IAA.

Indole acetic acid in the culture medium was quantified using Salkowski reagent. The absorbance of indole acetic acid (IAA) was measured at 530 nm, and the culture extracts contained varying amounts of IAA. The maximum concentration of indole acetic acid (60µgml-1) was found in broths supplemented with tryptophan for endophyte PDR7, whereas endophyte PDL1 had the lowest IAA content (24µgml-1). However, as illustrated in Figure 2, IAA was either undetectable or insignificant in the remaining fungal broth, both with and without tryptophan. The DNA extracts of all isolated endophytic fungi were subjected to *IaaM* gene amplification. In this regard, *Fusarium* sp. specific primers for the *IaaM* gene were used to amplify the *IaaM* gene present in the endophytic fungi. The partial amplified DNA fragment was approximately 400 bp. The amplified PCR products were purified, sequenced, and deposited in the NCBI database under GenBank accession numbers (Table 2).

**Table 2.** Five endophytic fungi and their *IaaM* GenBank accession number.

Isolates	Taxon	GenBank Accession no. (IaaM gene)
CDS11*	*Fusarium s*p.	MK281637
PDL1**	*Fusarium s*p.	MK281638
PDL3**	*Fusarium s*p.	PQ067510
PDR6***	*Fusarium s*p.	PQ014737
PDR7***	*Fusarium s*p.	PQ067510

*CDS ‘CD’ represents CDA medium, ‘S’ Stem.

**PDL represents the PDA medium, ‘L’leaf section.

***PDR represents ‘PD’ PDA medium, ‘R’ root.

### Germination and protocorm development experiment

**Figure 3.**
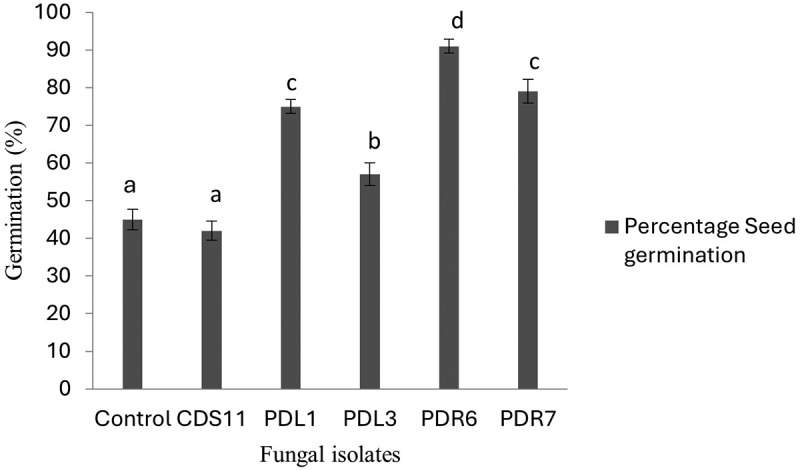
Germination response in seeds of *Dendrobium moschatum*. Supplementation with the extract of the isolated PDR6 yielded the highest percentage of seed germination as compared to other treatments. Bar represents mean ± SE (*n*= 15). p*≤0.05.*

**Figure 4.**
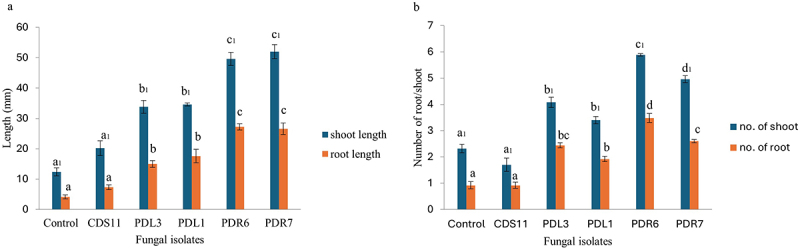
The protocorm development assay showing the effect of fungal extract in protocorm development of *Dendrobium longicornu*. The growth pattern treated with 2% fungal extract of PDR6 and PDR7 is higher in terms of mean of root and shoot length (a) as well as mean of roots and shoots number (b). Bar represents mean ± SE (*n*= 15). The data is significant at the level of *p*≤.05.

**Figure 5.**
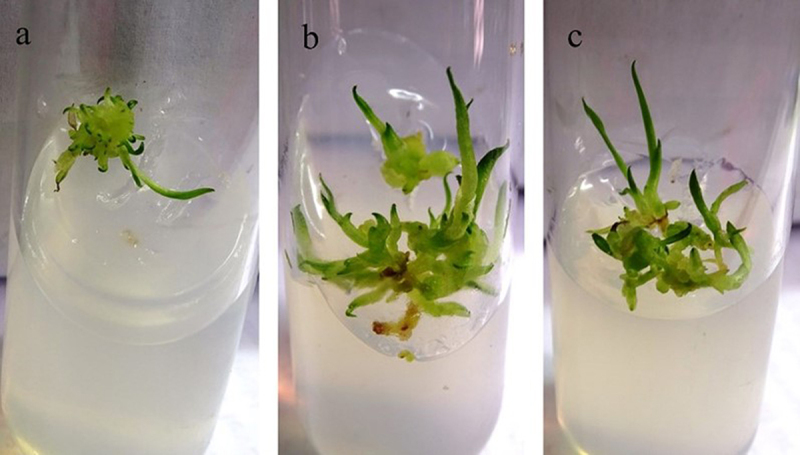
*In-vitro* plant growth assay of *Dendrobium longicornu* grown (a) on Murashige & Skoog (MS) media (control), or in MS media supplemented with fungal extract: (b) PDR6, (c) PDR7.

The effect of the fungal extract on seed germination percentage was measured and compared with that of the control. The seed germination assay was kept for ten weeks until the seeds turned green (Figure 3). The significance level of the seed germination assay was set at p < .05. The fungal extracts of isolates PDR6 and PDR7 were highly effective at approximately 80 and 90% (respectively) for seed germination. The plant growth assay was performed with protocorms of *D. longicornu* and extracts prepared from the fungal isolates CDS11, PDR6, PDR7, PDL3, and PDL1. The protocorms were allowed to grow the MS media containing 2% of fungal extract for seven weeks to observe the growth of the protocorms (Figure 4). The protocorms treated with fungal extracts of PDR6 and PDR7 in this experiment showed enhanced growth in terms of length and number of roots and shoots. Both PDR6 and PDR7 showed a five-fold increase in root length and a four-fold increase in shoot length compared to the control. Comparatively, PDR6 and PDR7 both had two- and three-fold increases in the number of roots, and both showed a five-fold increase in the number of shoots. Whereas CDS11 showed the least growth (Figure 5). The significance level of the protocorms development assay was set at p < .05.

## Discussion

*Fusarium* is an important group of non-mycorrhizal orchid fungi that have been reported as endophytes or pathogens based on the type of host tissue (healthy or diseased) [27,28]. They have been reported to originate from the roots, stems, and leaves of several orchids and are known to stimulate seed germination and plant growth [35–40]. *Fusarium* sp. was the first non-Rhizoctonia-like fungus reported to show a symbiotic association with orchid species [41]. Moreover, it is an orchid endophyte known to produce active metabolites in the host orchid [42–45] Crous et al 2019. Recent studies have shown that *Fusarium* is a common endophyte isolated from *Dendrobium* species [46]. A previous study indicated that *Fusarium* which may be a pathogenic or orchid-associated endophyte, may have a distinctive interaction with *Dendrobium* spp [47]. *Dendrobium nobile* and *D. moschatum* were infected with *Fusarium proliferatum* NRRL and *F. fujikuroi* IMI58289. *F. proliferatum* ET1 did not show any fungal infection. In addition, the plants remained healthy and asymptomatic which indicates the endophytic and beneficial nature of *Fusarium* species isolated from healthy plant material. Previous studies have shown that *Fusarium* sp. extracts isolated from *D. longicornuu* and *Vanda cristata* significantly promote the growth of in vitro-grown protocorms of *Cymbidium* and *D. longicornu* [14,15]. However, we cannot deny the pathogenicity of *Fusarium* species in orchids species. There are several reports regarding leaf-blight disease in orchids [48]. *Fusarium* and any other endophytes may become pathogenic depending on the plant’s age, immunity, abiotic stress or nutrient deficient condition [49,50].

Overall, the present study showed that most of the isolates were able to produce indole acetic acid as well as enhance in vitro plant growth and development. IAA production may be correlated with the coupling effect of two genes, *IaaM* (encoding tryptophan monooxygenase) and *IaaH* (indole-3-acetamide hydrolase) of the IAM pathway. This pathway has been reported to be fully functional only in orchid endophytes, especially *Fusarium* sp [26,51]. The fungal extracts of these isolates promoted growth of in vitro grown seeds of the host, as well as protocorm development of non-host orchids.

## Data Availability

The authors confirm that the data supporting the findings of this study are available in the article. The datasets analyzed in this study are accessible from the corresponding author upon reasonable request.

## Declarations

The field trip for sample collection was done in July 2018, as per the official guidelines provided by the Central Department of Botany, Tribhuvan University, Nepal. Dr. Keshav Raj Rajbhandri, botanist of National Herbarium and Plant Laboratories, Kathmandu, identified the plant. A voucher specimen of *Dendrobium longicornu*, voucher number N05, was deposited in the Tribhuvan University Central Herbarium (TUCH).

## References

[1] Shrestha KK, Bhandari P, Bhattarai S. Plants of Nepal (gymnosperms and angiosperms). Heritage Publishers & Distributors Pvt. Limited; 2022.

[2] Rokaya MB, Raskoti BB, Timsina B, et al. An annotated checklist of the orchids of Nepal. Nord J Bot. 2013;31(5):511–9. doi: 10.1111/j.1756-1051.2013.01230.x

[3] Pant B. Medicinal orchids and their uses: tissue culture a potential alternative for conservation. Afr J Plant Sci. 2013;7(10):448–467. doi: 10.5897/AJPS2013.1031

[4] Pant B, Shah S, Shrestha R, et al. An overview on orchid endophytes. In: Varma A, Prasad R, Tuteja N, editors. Mycorrhiza-Nutrient Uptake, Biocontrol, Ecorestoration. 2017. p. 503–524. doi: 10.1007/978-3-319-68867-1_26

[5] Pradhan BM, Bajracharya DM. Anatomical study of *Dendrobium* (Orchidaceae) of Nepal. Ann. Plant Sci. 2020;9(7):3916–3948.

[6] Hlosrichok A, Sumkhemthong S, Sritularak B, et al. A bibenzyl from *Dendrobium ellipsophyllum* induces apoptosis in human lung cancer cells. J Nat Med. 2018;72(3):615–625. doi: 10.1007/s11418-018-1186-x 29488156

[7] Paudel MR, Chand MB, Pant B, et al. Antioxidant and cytotoxic activities of *Dendrobium moniliforme* extracts and the detection of related compounds by GC-MS. BMC Complement Altern Med. 2018;18(1):134. doi: 10.1186/s12906-018-2197-6 29685150 PMC5913799

[8] Shrestha R, Sujit S, Bijaya P. Identification of endophytic fungi from roots of two *Dendrobium* species and evaluation of their antibacterial property. Afr J Microbiol Res. 2018;12(29):697–704. doi: 10.5897/AJMR2018.8924

[9] Pant B, Joshi PR, Maharjan S, et al. Comparative cytotoxic activity of wild harvested stems and *in vitro-*raised protocorms of *Dendrobium chryseum* Rolfe in human cervical carcinoma and glioblastoma cell lines. Adv Pharmacol Pharm Sci. 2021;2021:1–8. doi: 10.1155/2021/8839728 PMC7808824 33506210

[10] della Cuna Fs R, Boselli C, Papetti A, et al. Composition of volatile fraction from inflorescences and leaves of *Dendrobium moschatum* (Orchidaceae). Natural Product Communications. 2018;13(1):1934578X1801300127. doi: 10.1177/1934578X1801300127

[11] Thapa Magar MS, Maharjan S, Pathak J, et al. DNA barcoding of dendrobium moschatum (banks) sw. Specimen from Makawanpur, Central Nepal. J Pl Reso. 2022;20(2):124–129. doi: 10.3126/bdpr.v20i2.57001

[12] Pant B, Pradhan S, Paudel MR, et al. Various culture techniques for the mass propagation of medicinal orchids from Nepal. Acta Hortic. 2018;(1262):109–124. doi: 10.17660/ActaHortic.2019.1262.16

[13] Shah S, Thapa BB, Chand K, et al. *Piriformospora indica* promotes the growth of the in-vitro-raised *cymbidium aloifolium* plantlet and their acclimatization. Plant Signal Behav. 2019;14(6):1596716. doi: 10.1080/15592324.2019.1596716 30990122 PMC6546142

[14] Shah S, Shah B, Sharma R, et al. Colonization with non-mycorrhizal culturable endophytic fungi enhances orchid growth and indole acetic acid production. BMC Micrbiol. 2022;22(1):101. doi: 10.1186/s12866-022-02507-z PMC9006483 35418028

[15] Chand K, Shah S, Sharma J, et al. Isolation, characterization, and plant growth-promoting activities of endophytic fungi from a wild orchid *Vanda cristata*. Plant Signal Behav. 2020;15(5):1744294. doi: 10.1080/15592324.2020.1744294 32208892 PMC7238887

[16] Bhantana P, Rana MS, Sun XC, et al. Arbuscular mycorrhizal fungi and its major role in plant growth, zinc nutrition, phosphorous regulation and phytoremediation. Symbiosis. 2021;84(1):19–37. doi: 10.1007/s13199-021-00756-6

[17] Selosse MA, Petrolli R, Mujica MI, et al. The waiting room hypothesis revisited by orchids: were orchid mycorrhizal fungi recruited among root endophytes? Ann Bot. 2022;129(3):259–270. doi: 10.1093/aob/mcab134 34718377 PMC8835631

[18] Rekadwad BN, González Grau JM, Shah S, et al. Microbiome perspective: multisectorial exploitations of chitinases. Bentham Science Publishers. 2022. doi: 10.2174/9789815040340122020016

[19] Kashyap AK, Shah S, Pant KK, et al. Metabolomics and genomics for understanding stress biology of plant metabolites. In: Swamy MK, Kumar A, editors. Phytochemical genomics: plant metabolomics and medicinal plant genomics. Springer Nature Singapore; 2023. p. 629–649.

[20] Kashyap AK, Dubey SK, Shah S, et al. A short review on genes regulating biosynthesis of Major secondary metabolites. Phytochem Gen Plant Metabol Med Plant Gen. 2023;1:501–519.

[21] Thakur M, Khushboo SS, Kumari P, et al. Unlocking the secrets of rhizosphere microbes: a new dimension for agriculture. Symbiosis. 2024;92(3):305–322. doi: 10.1007/s13199-024-00980-w

[22] Zhao Y. Auxin biosynthesis and its role in plant development. Annu Rev Plant Biol. 2010;61(1):49–64. doi: 10.1146/annurev-arplant-042809-112308 20192736 PMC3070418

[23] Spaepen S, Vanderleyden J, Remans R. Indole-3-acetic acid in microbial and microorganism-plant signaling, FEMS microbiol. FEMS Microbiol Rev. 2007;31(4):425–448. doi: 10.1111/j.1574-6976.2007.00072.x 17509086

[24] Zhao Y. Auxin biosynthesis: a simple two-step pathway converts tryptophan to indole-3-acetic acid in plants. Mol Plant. 2012;5(2):334–338. doi: 10.1093/mp/ssr104 22155950 PMC3309920

[25] Fu SF, Wei JY, Chen HW, et al. Indole-3-acetic acid: a widespread physiological code in interactions of fungi with other organisms. Plant Signal Behav. 2015;10(8):e1048052. doi: 10.1080/15592324.2015.1048052 26179718 PMC4623019

[26] Tsavkelova E, Oeser B, Oren-Young L, et al. Identification and functional characterization of indole-3-acetamide-mediated IAA biosynthesis in plant-associated *Fusarium* species. Fungal Genet Biol. 2012;49(1):48–57. doi: 10.1016/j.fgb.2011.10.005 22079545

[27] Bhatti SK, Thakur M. An overview on orchids and their interaction with endophytes. Bot Rev. 2022;88(4):485–504. doi: 10.1007/s12229-022-09275-5

[28] Shah S, Chand K, Rekadwad B, et al. A prospectus of plant growth promoting endophytic bacterium from orchid (*Vanda cristata*). BMC Biotech. 2021;21(1):1–9. doi: 10.1186/s12896-021-00676-9 PMC7901085 33618710

[29] Katoh K, Misawa K, Kuma K, et al. MAFFT: a novel method for rapid multiple sequence alignment based on fast Fourier transform. Nucleic Acids Res. 2002;30(14):3059–3066. doi: 10.1093/nar/gkf436 12136088 PMC135756

[30] Capella-Gutiérrez S, Silla-Martinez JM, Gabaldòn T. trimAl: a tool for automated alignment trimming in large-scale phylogenetic analyses. Bioinformatics. 2009;25(15):1972–1973. doi: 10.1093/bioinformatics/btp348 19505945 PMC2712344

[31] Tamura K, Stecher G, Kumar S, et al. MEGA 11: molecular evolutionary genetics analysis version 11. Mol Biol Evol. 2021;38(7):3022–3027. doi: 10.1093/molbev/msab120 33892491 PMC8233496

[32] Khan AL, Halo BA, Elyassi A, et al. Indole acetic acid and ACC deaminase from endophytic bacteria improves the growth of *Solanum lycopersicum*. Electron J Biotechnol. 2016;21:58–64. doi: 10.1016/j.ejbt.2016.02.001

[33] Shah S, Shrestha R, Maharjan S, et al. Isolation and characterization of plant growth-promoting endophytic fungi from the roots of *Dendrobium moniliforme*. Plants. 2019;8(1):5. doi: 10.3390/plants8010005 PMC6359427 30597827

[34] Pradhan S, Tiruwa B, Subedee BR, et al. In vitro germination and propagation of a threatened medicinal orchid, *cymbidium aloifolium* (L.) sw. through artificial seed. Asian Pac J Trop Biomed. 2014;4(12):971–976. doi: 10.12980/APJTB.4.2014APJTB-2014-0369

[35] Srivastava S, Kadooka C, Uchida JY. *Fusarium* species as pathogen on orchids. Microbiol Res. 2018;207:188–195. doi: 10.1016/j.micres.2017.12.002 29458853

[36] Sarsaiya S, Shi J, Chen J. A comprehensive review on fungal endophytes and its dynamics on Orchidaceae plants: current research, challenges, and future possibilities. Bioengineered. 2019;10(1):316–334. doi: 10.1080/21655979.2019.1644854 31347943 PMC6682353

[37] Peschke HC, Volz PA. *Fusarium moniliforme* sheld. Association with species of orchids. Phytologia. 1978;40:347–356.

[38] Bayman P, Otero JT. Microbial endophytes of orchid roots. In: Schulz BJE, Boyle CJC, Sieber TN, editors. Microbial root endophytes. Berlin, Heidelberg: Springer Berlin Heidelberg; 2006. p. 153–177.

[39] Sufaati S, Agustini V, Suharno S. Short communication: *fusarium* as endophyte of some terrestrial orchid from Papua, Indonesia. Biodiversitas J Biolog Divers. 2016;17(1). doi: 10.13057/biodiv/d170149

[40] Maldonado GP, Yarzábal LA, Cevallos-Cevallos JM, et al. Root endophytic fungi promote in vitro seed germination in *Pleurothallis coriacardia* (Orchidaceae). Lankesteriana. 2020;20(1):107–119. doi: 10.15517/lank.v20i1.41472

[41] Vujanovic V, St-Arnaud M, Barabé D, et al. Viability testing of orchid seed and the promotion of colouration and germination. Ann Bot. 2000;86(1):79–86. doi: 10.1006/anbo.2000.1162

[42] Strobel GA. Rainforest endophytes and bioactive products. Critical reviews in biotechnology. Critical Rev Biotechnol. 2002;22(4):315–333. doi: 10.1080/07388550290789531 12487423

[43] Yuan ZL, Chen YC, Yang Y. Diverse non-mycorrhizal fungal endophytes inhabiting an epiphytic, medicinal orchid (*Dendrobium nobile*): estimation and characterization. World J Microbiol Biotechnol. 2009;25(2):295–303. doi: 10.1007/s11274-008-9893-1

[44] Crous PW, Carnegie AJ, Wingfield MJ, et al. Fungal planet description sheets: 868–950. Persoonia. 2019;42:291–473. doi: 10.3767/persoonia.2019.42.11 31551622 PMC6712538

[45] Hou XQ, Guo SX. Interaction between a dark septate endophytic isolate from *Dendrobium* sp. and roots of D. nobile seedlings. J Integr Plant Biol. 2009;51(4):374–381. doi: 10.1111/j.1744-7909.2008.00777.x 21452589

[46] Hajong S, Kapoor R. An amalgam of pathogenic and beneficial endophytic fungi colonizing four *Dendrobium* species from Meghalaya, India. J Basic Microbiol. 2020;60(5):415–423. doi: 10.1002/jobm.201900631 32115755

[47] Tsavkelova EA, Kolomeitseva GL. *Fusarium*–orchid interactions under greenhouse conditions. S Afr J Bot. 2022;146:889–896. doi: 10.1016/j.sajb.2022.03.038

[48] Yang J, Ahmed W, Zhang J, et al. Identification of *Fusarium oxysporum* causing leaf blight on *Dendrobium chrysotoxum* in Yunnan Province, China. Life. 2024;14(3):285. doi: 10.3390/life14030285 38541611 PMC10970817

[49] Redkar A, Sabale M, Zuccaro A, et al. Determinants of endophytic and pathogenic lifestyle in root colonizing fungi. Curr Opin Plant Biol. 2022;67:102226. doi: 10.1016/j.pbi.2022.102226 35526366

[50] Collinge DB, Jensen B, J JH. Fungal endophytes in plants and their relationship to plant disease. Curr Opin Microbiol. 2022;69:102177. doi: 10.1016/j.mib.2022.102177 35870225

[51] Tang J, Li Y, Zhang L, et al. Biosynthetic pathways and functions of indole-3-acetic acid in microorganisms. Microorganisms. 2023;11(8):2077. doi: 10.3390/microorganisms11082077 37630637 PMC10459833

